# Combining Segmentation and Edge Detection for Efficient Ore Grain Detection in an Electromagnetic Mill Classification System

**DOI:** 10.3390/s19081805

**Published:** 2019-04-15

**Authors:** Sebastian Budzan, Dariusz Buchczik, Marek Pawełczyk, Jiří Tůma

**Affiliations:** 1 Institute of Automatic Control, Silesian University of Technology, Akademicka 16, 44-100 Gliwice, Poland; dariusz.buchczik@polsl.pl (D.B.); marek.pawelczyk@polsl.pl (M.P.); 2 Department of Control Systems and Instrumentation, VŠB-Technical University of Ostrava, 17. listopadu 15/2172, 708 33 Ostrava-Poruba, Czech Republic; jiri.tuma@vsb.cz

**Keywords:** grain detection, seeded region growing segmentation, edge detection, feature extraction

## Abstract

This paper presents a machine vision method for detection and classification of copper ore grains. We proposed a new method that combines both seeded regions growing segmentation and edge detection, where region growing is limited only to grain boundaries. First, a 2D Fast Fourier Transform (2DFFT) and Gray-Level Co-occurrence Matrix (GLCM) are calculated to improve the detection results and processing time by eliminating poor quality samples. Next, detection of copper ore grains is performed, based on region growing, improved by the first and second derivatives with a modified Niblack’s theory and a threshold selection method. Finally, all the detected grains are characterized by a set of shape features, which are used to classify the grains into separate fractions. The efficiency of the algorithm was evaluated with real copper ore samples of known granularity. The proposed method generates information on different granularity fractions at a time with a number of grain shape features.

## 1. Introduction

Grain detection plays an important role in the grinding process of ore minerals. When taking into account the different features of ground material, the size and shape of the grain are important indicators of a high-quality grinding process. Active work in the field of grain detection optimization is important because a wide range of industrial applications engage grinding as a vital operational input. Grinding is invaluable in carbon steel microstructures [1], metal ore milling [2], cement production [3], minerals [4], powder technology for the food industry [5], and the pharmaceutical industry [6], among others. Based on final particle size, time consumption, parameters of the grinding process, grinding media, assumed particle size, and type of material used, we identify various types of mills that have described in recent literature—the ball [7], vertical roller [8], electromagnetic [9,10], pin mill [11], Buhrstone [12], and others. 

The grinding process is based on disintegration of the material by different kinds of grinding media. An inappropriate grinding process—grinding to a smaller size than necessary or too coarse milling—increases energy consumption and media demand, and decreases recovery of key material (such as copper from copper ore) and overall grinding efficiency. Thus, it is important to dynamically evaluate the entire grinding process in a way that is as objective as possible. Based on a different construction of mills, different methods of overall quality evaluation can be applied. In the electromagnetic mill, the speed of grinding material and chaotic movement of material and rods in the working chamber are the main reasons of real-time application development, instead of ball mills, where the final granularity of the material is reached in minutes, not seconds. Thus, we focused on the real-time processing of the final product, which is one of the major novelty of the proposed method. However, at times, faster evaluation is required, such as in the case of detected defects in the grinding process, which may be the result of incorrect control, defects in mill components, or errors in online analysis.

One of the most useful methods of evaluating grinding quality is based on grain/particle size and shape analysis. This method has its basics in technical diagnostics, wherein analysis of the final product is made. Because grinding is a disintegration process, grain size gradually decreases; in addition, grain shape also changes rapidly depending on material type, e.g., sand grains round off much faster than copper ore grains. The contribution of smaller grains during grinding increase substantially, while that of the larger ones decrease. Thus, effective detection and classification of the grains in the tested sample of ground material plays an important role in the mill’s control system. 

Detection and analysis of grain size with shape is challenging and time consuming. Thus, there is the need to develop a fast and effective method that accurately determines grain size. Image processing particle recognition methods are rapidly growing grain detection techniques with a wide range of industrial applications. These methods have varying ways of acquiring data, such as Outotec Sympatec solutions (based on laser diffraction), Comex (based on X-ray); and others that engage 2D/3D laser scanning, near infrared (NIR), infrared (IR), and vibration. 

Image-based methods can be divided into two different approaches: Edge and Region. The first category can be applied for images with high quality, contrast and depth; where neighboring boundaries can be easily extracted from the image. In most cases, images taken directly from the grinding process have poor edges. The second method focusses on segmentation by region growing. Grains detection and classification can be performed on the basis of geometric parameters such as area and diameter. However, color and texture is useful for classification, especially when the active surface of a metal ore is taken into account. In the proposed method we used computer vision laboratory stand, which is based on acquiring images of samples taken strictly from the rig and gravitationally transported on the material slope to the working field of view of the camera. The method of transportation can be a reason for poor samples, thus, the authors developed a method based on proper illumination and aperture control for extraction of the metal surfaces from ore grains, also pre-processing algorithm.

In previous studies, we observed incorrect detection of grains in boundary areas and detection of false grains due to merging of smaller grain areas during segmentation. Despite application of the adaptive criteria of homogeneity, we obtained a significant number of grains that required further processing and division into smaller sizes on completion of the main segmentation. An important element that we took into account is the spectrum of application of the developed method. We focused on the correct detection and classification of grains to assess the grinding level of the valuable material. Our method also allowed us to detect the active surface of copper ore and to analyze the dependence of grinding quality on material moisture, which is important during wet grinding. We proposed a method that combined both of the mentioned approaches—region segmentation and edge detection—where region growing is limited only to the boundaries detected in the edges of the image. 

The process starts with a region growing from the seed until reaches the first pixel of the grain boundary and switches to the edge tracking. The main problem connected with segmentation is a homogeneity criteria and a threshold value. In most cases, threshold is constant with predefined value or depends on local or global information from the image according to segmentation method. We decided to use a modified thresholding technique, which utilizes global and local information by the use of intensity and derivatives of the image, which simplifies the problem of region growing with boundaries information, extracted from the grains’ images. One of the main advantages of our method is its wide range of applications: detection and tracking of selected fractions with large grains, detection and tracking of a few fractions with different grains sizes, detection of the active surface of copper ore, and ascertaining the relationship between material moisture and granularity. The paper proposes the following novel contributions:

- An efficient real-time machine vision-based method of grain detection and classification of different sizes, metal ore, scales, and shapes using an electromagnetic mill system
- An improved method of sample quality checking based on cascade of 2DFFT and GLCM
- A new combined SRG with boundary information using edge detection
- A new thresholding procedure with a threshold calculation of homogeneity, which analyzes the possibility of applying the method to different applications

The paper is organized as follows: Section 2 presents a detailed description of the state-of-the-art methods using hardware and algorithms; Section 3 describes the proposed methods, including hardware setup, image acquisition, pre-processing of modules, and grain detection and classification with calculation of shape features; Section 4 presents a discussion, authors’ remarks, and results. Conclusions and future works are drawn and presented in Section 5.

## 2. Related Work

The choice of evaluation method of material granularity depends, to a large extent, on the type of mill that is used. In the case of a ball mill, whose grinding times are much larger than an electromagnetic mill, especially in the first part of the grinding, when the large grains of feed material is ground by the rods, instead of the further part, where grains are ground by the rods and collisions with other grains. Generally, for a one selected granularity of the final product in µm we can get the same final granularity in second, instead of in minutes for ball mill. For a ball mill which operates in minutes, a laboratory test can be used. For an electromagnetic mill, where grinding is counted in seconds, it is important to accurately and quickly evaluate granularity in real time. The choice of method is often determined by the minimum grain size to be detected; in other cases, it is identified by simultaneous detection of different fractions of the same material and detection of different materials at the same time, e.g., copper ore with dolomite. The purpose of granularity fraction detection is important; the difference is when detection is a part of the analysis before the flotation process, compared to only grinding of the material to assume granularity. Other results can be achieved using laser diffraction, Scanning Electron Microscopy (SEM), machine vision methods, laboratory sieves, vibration measurement, NIR, IR, or X-ray. Recent literature offers numerous methods of grain parameter measurement, depending on material type in most cases. They are described by different time consumption measurements, detectable grain size range, and method of sample preparation—from the rig to the test stand. The semi- or fully-automatic grain parameter evaluation methods are divided into well-categorized groups, such as vibration screening technology, vibration measurements, acoustics, and optical methods.

A screening machine, also called a sieve machine, is the most popular method of separation of different fractions [13,14]. This method uses screening material of a certain weight and pore size to separate the grains and during the process moves on to finer sieve aperture screens. Each sieve has a specific percentage weight for each predefined fraction. Clogging is the main disadvantage of this technology, which, however, has been resolved to a certain extent with the introduction of a self-clogging feature in some excitations sieves. The second sieve is connected to first on the basis of the shape of the particle and sieve aperture (selected taking into consideration the maximum width and thickness of the particle) [15]. In some cases, very long and thin particles can be categorized as small. The classic screening machines require correct screening plate aperture that consider material type, size, and moisture. Thus, researchers placed focus on vibrating screens with reconfigurable or adjustable screen surface structures [16]. The authors of [17,18] adopted well-known vibration measurements to measure granularity of flowing material in a laboratory with six classes of granularity material. In this method, measured vibrations in predefined locations on the rig, were used to evaluate granularity and flow rate, based on the assumption that loose material in pipelines generate vibrations that are dependent on the granularity of the material. The main advantage of this method is the possibility of using small, inexpensive vibration sensors. A main problem, however, is related to the detection of smaller granularity fractions, because most of them generate similar vibration signals.

Optical methods process grains of different materials. On one hand, this technique, which is a rapidly growing mechanism, offers the ability to process images obtained in a wide spectral range (400 nm to 1 mm) [4], as well as enables combinations for multiresolution images [19]. On the other hand, it presents numerous possibilities to detect, recognize, and classify objects using colors, shapes, boundaries, texture, as well as detailed features such as physical, statistical, and dynamic changes in parameters values. In addition, image processing in a visible spectrum offers the possibility of processing the images in different color, time, and frequency domains. Grain detection is a challenging task with a wide range of applications, such as in nanoparticle detection with circular Hough Transform [20]; river-bed grain size determination based on neural fuzzy network [21]; segmentation of petrographic thin section images [22]; characterization of particulate minerals such as celestite with inexpensive machine vision system [23]; monitoring of industrial flotation cells in an iron flotation plant [24]; separation of different ores and gangue minerals with multicriteria decision making [25]; refinement of raw plain carbon steel microstructure images [26]; and particle size distribution of ball-and gyro-milled lignite and hard coal [27]. 

The choice of processing method of digital images for the detection of grains depends on the shape of the grains. If they are of regular shape, pattern detection methods can be used based; for example, the Hough Transform [28]. However, if the shapes are irregular, such in copper ore, two methods are usually employed: edge-based methods (boundaries between grain detection) [29], and region-based (region growing segmentation) [30]. Edge-based methods lean on processing of the gradient images with morphological operations such as opening, closing, thickening, and skeletonization. In [31], the authors proposed a new method to improve rock classification with feature selection based on mutual information and boundary information. The segmentation method, on the other hand, uses the constrained automated seeded region growing technique [32]. A hierarchical neuro-fuzzy model for classification of macroscopic rock texture has been proposed in [33]. Color features have been widely used in [34] by Obara, where space transformation from RGB to CIElab was proposed. Color and textural features have been considered in [35] with Principal Component Analysis (PCA) and Wavelet Texture Analysis (WTA).

Finally, we developed, in our laboratory, certain processes of grain detection and classification with image processing for the copper ore grinding process. In [36], the authors propose a general method to extract information about the grains in the sample, based on Otsu segmentation with classification procedure, which is based on calculated shapes factors. The proposed method focuses on angle illumination, which improves contrast in the images, especially on the edges of the grains. The method described in [37] is focused on automatic seeded region segmentation, optimized by Relative Standard Deviation (RSD) calculation. Next, the quality of the detected grains was improved by a usage distance map, which separated the connected grains. Finally, classification of the grains was done based on calculation grain parameters such as perimeter, aspect ratio, and other shape features specified for metal ore grains. The proposed solution deals with a few main problems, such as selecting seed points, similarity criteria, and fixed or adaptive threshold connected to the similarity criteria.

## 3. Materials and Methods

Laboratory methods are intense and time-consuming—samples are collected from a technological line, prepared, and then analyzed using specific applications. Another aspect is the cost of the system, especially when methods such as X-ray and laser diffraction are used. Different methods offer varying information on the grains, i.e., color, shape, size, type of mineral, share of metal in the ore. In this research, we wanted to determine the level of grinding that could be achieved and to be able to assess the amount of active surface of copper. A machine vision system, thus, was found to be a natural choice and optimal solution. We were able to achieve a level of 80–100 µm of grinding material. The proposed system can extract several types of grains features, which include color, texture, and morphology. The complexity of the copper ore grain structure (localized at different positions and angles) in the prepared sample could result in false outcomes. Thus, we decided to use different features extracted from the spatial and frequency domain to identify texture and morphology (grain shape and size), respectively, in a different step of the algorithm.

The grain size analysis methods described in the previous section have advantages and weaknesses; however, in order to determine the distribution of granularity fractions, we made several assumptions, which allowed us to choose one method of analysis:

- The analysis should be done at the mill stand, not in a laboratory
- The system should be inexpensive in comparison to X-ray or laser diffractometry
- The range of detected grain fractions should be as wide as possible
- Analysis time should be as short as possible, mainly due to short grinding time in an electromagnetic mill, mostly lasting seconds
- The analysis should allow grain shape features to be obtained at a given time

**Figure 1:**
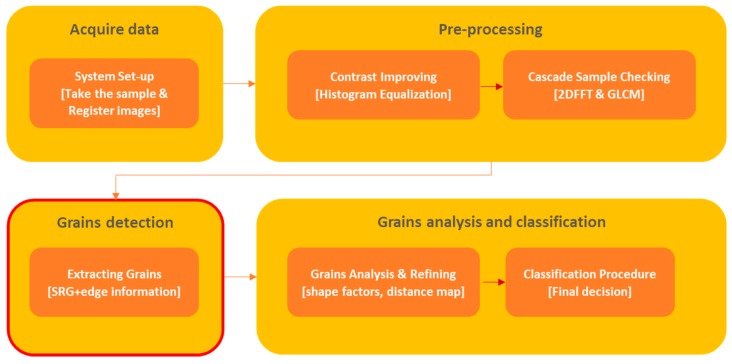
Block diagram of the proposed method.

Our proposed method for copper ore grain detection and classification contains a few well-defined steps, as presented in Figure 1. First, the ground material sample is collected from the rig and gravitationally transported to the vision system. Next, the quality of the sample is improved by contrast enhancing. The sample is also tested with a two-stage cascade classifier: in the first stage, we performed a coarse test by using 2DFFT transform to check uniformity of the sample; in the second stage, a detailed test was performed using the GLCM. When the sample passes this quality test, combined SRG with boundary information for detection of grains is carried out. We decided to combine these regions and use edge-based technique with additional illumination for better extraction of grains boundaries. Image processing however, was based only on modified SRG, without information about boundaries. Following this step, shape features for all of the detected grains were calculated. This distance map-based method enhanced the final number and shape of the detected grains. Finally, based on the identified shape features, a simple voting process classified grains into defined fractions with a final decision on grinding process quality.

### 3.1. Electromagnetic Mill Construction and Sample Preparation

The research and method described in this paper were performed with an optimized electromagnetic mill, developed and constructed at the Silesian University of Technology. A detailed description of the working principle, measurement, and control system can be found in [38]. In this mill, material is ground by ferromagnetic grinding media (rods) in a working chamber. The main idea assumes that the mill structure and vertical position of the working chamber, which is filled with material from the top and a stream of transport air from the bottom, is stationary during the grinding process. The generated rotating electromagnetic field moves the grinding media and the feed material is ground by numerous collisions between rods and processed material’s grains. The size of the rods depends on the mill working chamber diameter and a feed, in most cases 1–3 mm of diameter and 10–15 mm of length. The grinding media moves in a chaotic manner inside the working chamber which increases the grinding process. The electromagnetic mill performance depends on specific roads size, operating frequency on the inducted field, material flow rate, material moisture. In comparison to the ball mill, electromagnetic mill requires specific grinding and classification rig with the integrated control system. The developed mill provides a significant reduction in energy consumption and a higher level of technological performance, effectively producing particles with specific parameters, especially with the desired shape such as sharp grain edges or more cubic ones with soft edges regarding the different granularity of the ground material.

**Figure 2:**
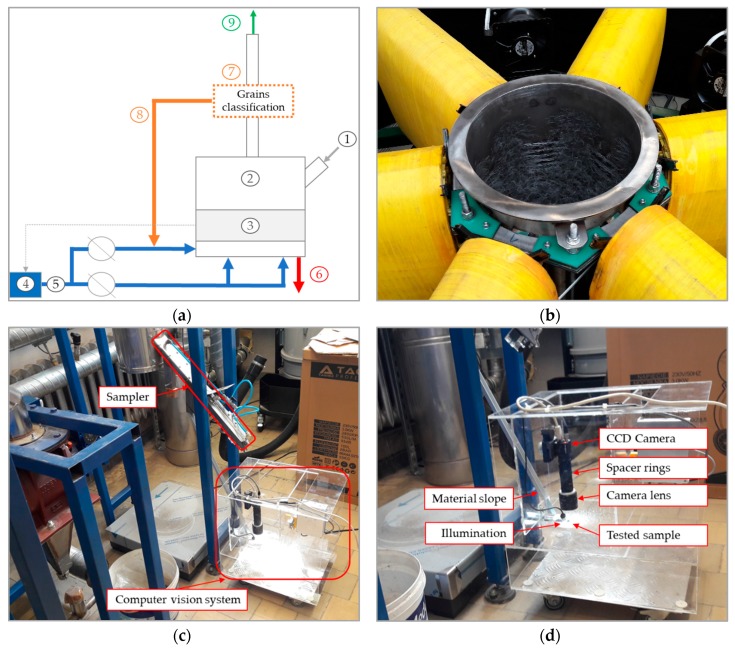
Schematic representation of the electromagnetic mill (**a**), working chamber with visible grinding media (**b**), a part of electromagnetic mill with sampler and computer vision system (**c**), the laboratory stand with computer vision system (**d**).

In Figure 2a, a schematic representation of the electromagnetic mill is provided, which includes components such as stream of the feed material (1); working chamber with electromagnetic field inductors and a preliminary classifier (2); working space with the ground material and the grinding media (3); source of air (4); main and additional air stream (5); stream of material that was not ground (6); machine vision grain detection and classification module (7); recycle stream of greater-than-assumed particle size (8); and final product (9), which moves to the cyclone and finally on to the final product tank. In Figure 2b, the working chamber with moving in electromagnetic field rods is presented. In contrast to grinding with the feed material in this case rods moving on specific vertical lines, except some chaotic collisions. It can be controlled among other methods by correct selection of induction level, number, and position of induction windings.

The feed material was of variable size in the range of 0–2 mm and a minimum of 45 µm as final product. We decided to set the vision system with a minimum required parameters in a wide range of granularities such as camera resolution and lens. In another case, these parameters can be suited to the feed material granularity or target granularity product. Besides the type of dry or wet milling should be taken into account. Results in this paper have been obtained only for dry milling. Our method was tested on a computer vision laboratory stand selected according to the mentioned issues. The system is equipped with a 1624 × 1234 and 2448 × 2048 resolution monochromatic CCD 2/3”camera with global shutter, lens with 50 (49.93) mm focal length and F1.8-16 aperture, extension tube with set of spacer rings, max. 0.5, 1, 2, 5, 10, 20, 40 mm, one and two LED sources of co-axial spot white light with Ø6 mm diameter and a personal computer with developed software for control camera with illumination and grains detection and classification. In Figure 2c the laboratory stand with the sampler connected to the mill rig is presented. The material is sampled with a predefined frequency directly from a rig and then transported gravitationally with a simple material slope strictly to the field of view of the camera. The detailed laboratory vision stand is presented on Figure 2d. The proposed 5 MP resolution CCD camera and combination of spacer rings and lens for 1 cm^2^ field of view will acquire and possible detection of grain size in the range below 100 μm, whereas for grains with a size of 80 μm, we have about 20 pixels, and for grains of about 45 μm, only 12, where evaluation of correct and real shape is a definitively hard, because the 12 pixels grains can be homogeneous or strictly rugged.

### 3.2. Pre-Processing Algorithm

**Figure 3:**
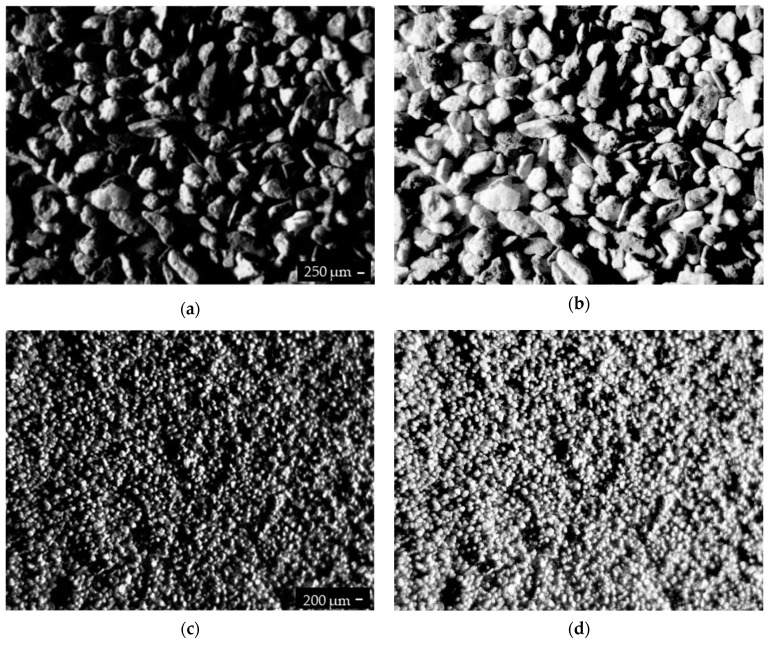
The machine vision system acquired image (**a**,**c**), images after HE (**b**,**d**).

Images of the copper ore samples were acquired by the vision system, which was equipped with a camera, two lights mounted on opposite sides, and a predefined camera axis angle between 30–45°; this illumination helped extract information on the edges and top of the grains. The acquired image contains valuable information (such as valleys/hills), which can be used to evaluate quality. Despite the angle illumination, we should deal with some low-luminance problems in the image. If the angle between illumination and sample will be small, the edges of the grains in the sample will be extracted in contrast to a planar region. Thus, the selected angle must be a compromise between overall image luminance and a number of extracted grains boundaries. In consequence, the pre-processing part of the algorithm was developed to improve detection results and processing time by eliminating not correctly prepared samples, before the main process is carried out. Figure 3a–c shows a low-luminance image, wherein detection of grain size and shape is incorrect. To correct this, image contrast must be enhanced through global and local approaches with respect to grain’s edges. The first approach improves image contrast by extending a dynamic range of intensity using the histogram of the complete image. The local approach, on the other hand, only uses local information inside each separated block. In some cases, the blocks overlap, but the intensity of the whole image is still omitted. Recent literature discusses numerous global and local methods of contrast enhancing, such as based on the bi-histogram equalization median plateau limit [39]; clipping the histogram, where maximum value of histogram is controlled by clipping histograms higher than the predefined threshold [40]; combination of Histogram Equalization (HE) and histogram clipping in exposure based sub-image histogram equalization [41]; and gradient-based local histogram equalization to preserve image texture [42]; among others. 

The authors of this paper used the HE algorithm, which enhances the finer details of low-luminance images. HE is an automatic and fast contrast improvement technique, which operates in the spatial domain on the pixel level of the image. HE changes the mean brightness of the input image to the middle level; in other words, it flattens the density distribution and stretches the dynamic range of gray levels in an image. Global contrast enhancement techniques have one main disadvantage—they increase or decrease contrast in an image at the same level, thus changing details of individual objects on simple, homogenous backgrounds. HE, on the other hand, boosts bright areas or removes dark regions in an image without difference to individual spots. This feature is clearly visible in complex scenes, where there is no uniform background and there exists numerous objects, or one larger object with numerous different items. Figure 3b,d presents the results of HE of different copper ore fractions. The images of a large number of grains were enhanced by eliminating dark areas and enhancing light ones.

In the next step, the uniformity of the sample surface is examined with the use of the conditional cascade method. The cascade is deemed conditional because the second cascade is only executed when the sample passes the first. We observed that the surface of the sample was not flat due to defects such as valleys and hills, which were created when the material moved from the rig to the vision system. There were several defects even when the sample was mechanically flattened. It was not possible to remove all the defects, but we were able to use samples with small ones. 

We used post-HE images as input, divided into four symmetrical, non-overlapping sub-regions. These sub-regions are processed separately in both cascades. As a result of our observations, we divided defects into two types based on their shape and size. The first type is usually visible in the dark areas of an image, which is associated with the formation of valleys of considerable size—the shape depends on the speed of material sliding, which directly depends on the granularity fraction. The regions of defects of this type are usually large in size and elongated in one direction—towards at least two neighboring sub-regions. In smaller granularity fractions, valleys have an irregular shape and are contained in a separate sub-region; however, they are often greater in number. The first type of defect is examined in the first cascade, which is based on the frequency domain with the use of fast Fourier transform. The second cascade, which is based on spatial information with the use of GLCM, detects a second type of defect.

**Figure 4:**
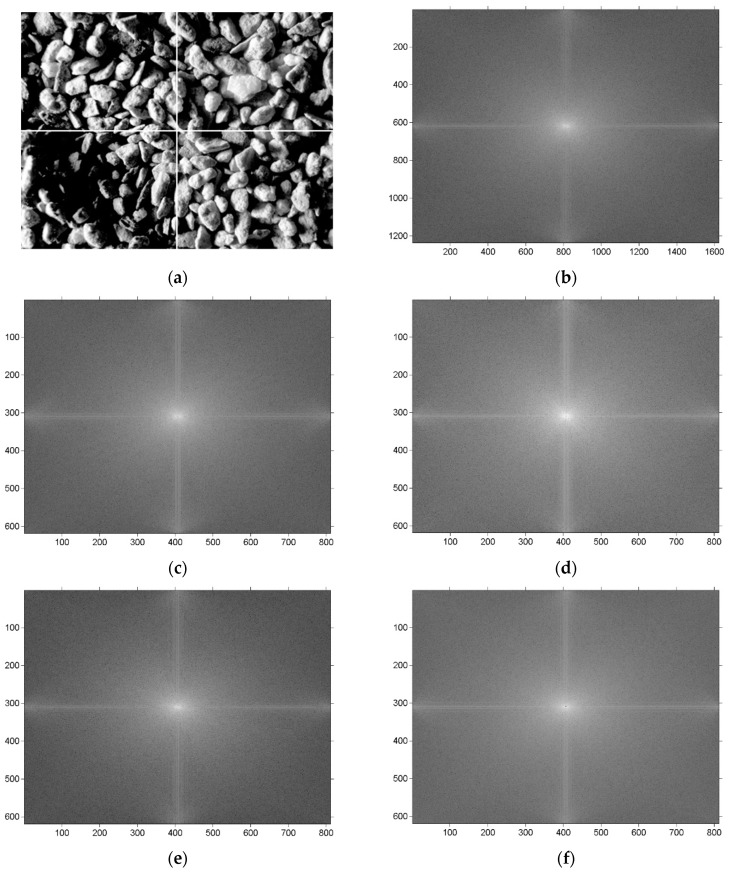
Image of the sample after HE with a marked sub-region (**a**), 2DFFT result (**b**), 2DFFT result for the top-left sub-region (**c**), 2DFFT result for the top-right sub-region (**d**), 2DFFT result for the bottom-left sub-region (**e**), 2DFFT result for the bottom-right sub-region (**f**).

The Fast Fourier Transform (FFT) [43] is a technique that offers a wide range of applications, such as image analysis, image filtering, motion control, and compression. In general, low frequencies represent smooth parts of the image with small variations, while high frequencies denote rapid changes at a gray level, such as contours, edges, and rough objects. FT can, thus, be used to detect large defects, because they have an effect on low frequencies. A classic FT calculation, which consists of 2DFFT, a power density spectrum, shift to the center, and logarithmic transformation, was performed for symmetrical, non-overlapping sub-regions in this study. Each sub-region was described by a mean magnitude. In most cases, the values differ; thus, we rejected samples wherein the largest difference between two out of four mean magnitudes was greater than 2%. If that was not the case, we examined the sample using the second cascade. Figure 4 shows a sample with the first type of defect. The bottom-left identifies a sub-region, large valley with a corresponding power spectrum (see Figure 4c,e), which differs visually for lower frequencies with the numerical difference between the mean magnitudes being 8.2–12.9%.

The second cascade extracts detailed information from the images. Interesting defects in this cascade are represented by small gray value regions of pixels, located as an irregular pattern on the image. In our previous research, we used a simple and fast method, which was based on the calculation of average intensity and standard deviation for each sub-region. These parameter values were compared with nominal sample parameter values; if the calculated values were less than 80% of the nominal ones, the sample was rejected. Unfortunately, average intensity and standard deviation do not fully describe image texture when there are a large number of grains. Thus, the GLCM method [44] was selected as a reliable method for the description of texture in complex images. This method calculates all the transactions between intensities at specified positions, relative to each other in the image. In other words, GLCM identifies how often a pixel with gray-level value *i* occurs either horizontally (0°), vertically (90°), diagonally bottom left to top right (−45°) or diagonally top left to bottom right (−135°) with regard to neighboring pixels with value *j*. The GLCM matrix dimension depends directly on the number of gray levels; thus, matrix dimensionality is reduced to the specified number of gray tones, due to a significant sensitivity for the size of the objects in the image. 

**Figure 5:**
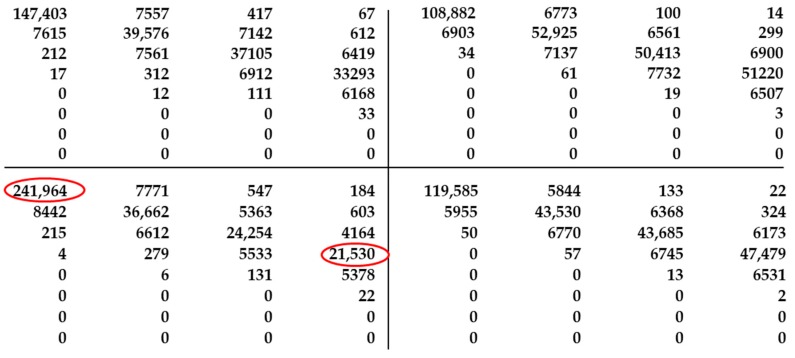
Calculated GLCM matrix for the top-left sub-region shown in Figure 4a.

The top-left value in Figure 5 corresponds to the number of transactions between the same grey levels 0. Thus, the bottom-right value is a result of transactions (3,3). A part of the GLCM matrix has been presented for sub-regions defined in Figure 4a. In the bottom-right sub-region, transitions (0,0) and (3,3) marked with red ellipse differ from similar transitions in other sub-regions. This observation is correct and in this case, has a direct relation to the real sample image. However, the GLCM method was used for less-distinctive defects. Thus, correct and in-depth information about texture in the image should be extracted from the GLCM matrix. Haralick proposed 14 different textural features for textural characteristics; we selected four, based on previous research: energy (*G_E_*), correlation (*G_C_*), inverse difference moment (*G_H_*), and variance (*σ*). The definition is presented in Equation (1):

(1) $$\begin{array}{cc} G_{E}=\overset{g-1}{\underset{n=0}{\sum }}P{\left(i,j\right)}^{2}, & G_{C}=\overset{g-1}{\underset{i=0}{\sum }}\overset{g-1}{\underset{j=0}{\sum }}P\left(i,j\right)\frac{\left(i-\mu \right)\left(j-\mu \right)}{\sigma^{2}}\\ G_{H}=\overset{g-1}{\underset{i=0}{\sum }}\overset{g-1}{\underset{j=0}{\sum }}\frac{P\left(i,j\right)}{1+{\left(i-j\right)}^{2}}, & \sigma^{2}=\overset{g-1}{\underset{i=0}{\sum }}\overset{g-1}{\underset{j=0}{\sum }}P\left(i,j\right){\left(i-\mu \right)}^{2} \end{array}$$

where *g* is a number of gray levels, *P*(*i*,*j*) is a pixel intensity, *µ* is a GLCM mean, *σ* is a variance of the intensities of all reference pixels in the relationships that contributed to the GLCM.

Energy is the sum of the squares of values in the GLCM, and is high for images with high homogeneity. Correlation measures the linear dependency of grey levels of neighboring pixels. The high local homogeneity (uniformity) of the image is extracted by inverse difference. Finally, the variance describes the contribution of individual pixel intensity in the image. The variance is smaller when the pixel’s similarity to its neighbors is greater. All the features calculated for each of the four sub-regions must be examined by the first cascade rule. If the features are similar in all four sub-regions, the image is processed using the grain detection procedure.

### 3.3. Grains Detection Algorithm

The main goal of this paper is effective detection and classification of separated grains of copper ore using images of the ground sample. Well-known object detection techniques can identify grains by their stationary features (surface color, shape, texture, and size), dynamic changes in shape and size, and indirect methods such as time taken to submerge in a liquid (large grains take lesser time than small ones). Our research takes into account a few vital requirements—wide range of detected grains fractions in one sample, especially the largest one; and image decision without any tracking, color/gray level and simultaneous shape analysis. 

The images acquired using a machine vision system show grains on the entire surface of the material, clearly visible by a camera; many of the separate grains have similar color or texture, despite the HE. Image segmentation is the right way to identify the grains, especially an adaptive method with region growing. Several algorithms have been proposed based on different uniformity criteria in other industries [45,46]. This approach, however, has a few main problems: selecting seed points, similarity criteria, correlation of the fixed or adaptive threshold with the similarity criteria.

Our previous research focused on modifying the Otsu method, which takes into account local changes in texture; it produced good results only for non-connected grains. The method is based on a growing region, starting from a point in the segmented region (e.g., grain), and proceeding to add objects that are similar to the seed, until all such points in the image are examined. The uniformity of the grains was determined using similarity criteria, which was calculated for the current region by using the well-known Relative Standard Deviation (RSD). RSD is defined as the ratio of standard deviation to the mean. In the proposed solution, RSD must be calculated each time for the current region and for the current region with a possible added point. If these two values are similar, then the tested point is added to the current grain. Of course, as a consequence, some of the detected grains are of different size. Each grain is described by a set of shape features—aspect ratio (*F_A_*), compactness factor (*Fc*), and Heywood circularity factor (*F_H_*)—which carry information about non-typical grains. They are defined as follows:

(2) $$F_{A}=\frac{d_{min}}{d_{max}},F_{C}=\frac{A^{2}}{2\pi \sqrt{i_{1}^{2}+i_{2}^{2}}},F_{H}=\frac{4\pi A}{P^{2}}$$

where *d_min_* and *d_max_* are the smallest and largest diameter respectively, *A* is a area, *i*_1_ and *i*_2_ are two second moments of the grain around its principal axes, *P* is a perimeter. 

**Figure 6:**
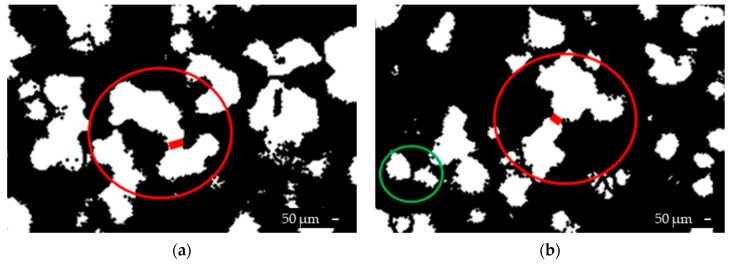
The images after the proposed segmentation with merged grains (**a**) and with both, correctly separated (green circle) and not (red circle) grains (**b**).

This study found the grains to have very low aspect ratio, low value of compactness, and a large value of Heywood factor. Next, the selected grain is divided into a few smaller ones, based on the calculated distance maps. In this method, a significant part of the work is done using distance map calculation, wherein more than 15% of the grains are restored. In most cases, the grains overlap or have a common boundary; thus, region growing is performed, producing merged grains. Figure 6 presents samples of the merged grains after the segmentation method. 

Separation of the various, adjacent homogeneous regions, based only on segmentation, is a crucial task. The reasons why segmentation needs to be improved are: to improve the number of parameters that must be selected (even if they are selected automatically), varying localizations of the grains, different grain sizes, different pixels intensity, and number of grains classified into fractions. 

**Figure 7:**
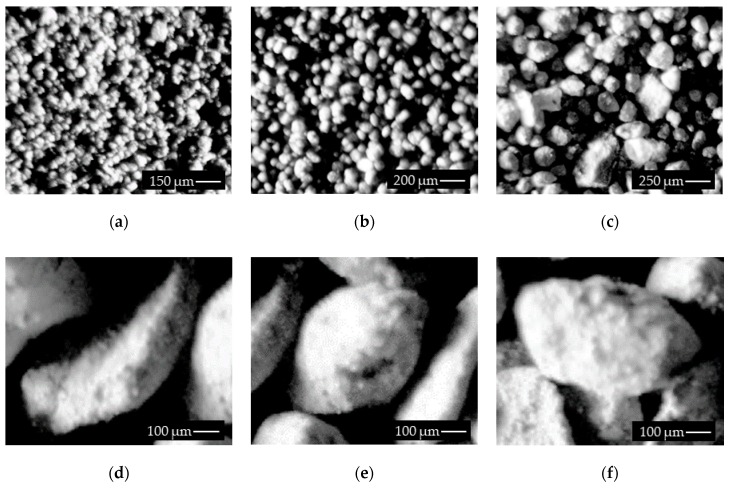
Real images of tested samples (**a**–**c**), grains with 0.75 mm granularity fraction (**d**–**f**).

The most challenging issue concerning copper ore detection from real images is correct region recognition for different fractions. In Figure 7a–c, granularity fractions in the range of 0.25–0.75 are presented at different positions, angles, and sizes. Furthermore, three common situations have been identified: a separated, elongated grain (Figure 7d); oval grain with a complex surface—bright and dark areas (Figure 7); and an intermediate shape with four connected grains (Figure 7f).

Based on this, we characterize grains as a non-uniform region, where pixel intensity has a wide range and varies in the region, edges that are not thin, one-pixel edges, and common boundary between grains. Based on human vision, which is sensitive to edges in the scene [47], previously identified SRG properties have been improved upon, with focus on region growing in combination with boundary information, extracted with the use of edge detection. 

**Figure 8:**
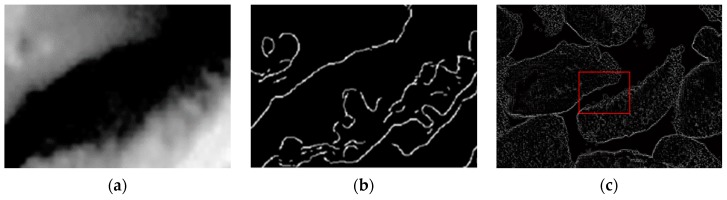
The original zoomed sample image of Figure 7d with two visible grains (**a**), corresponding edge image after Gaussian smoothing and Canny detection (**b**), calculated second derivative, part of (**a**) marked with red color (**c**).

Edge-based methods are suitable to detect step changes in the images, especially linear and points features. Figure 8a presents two grains from the same sample that show a significant difference between boundaries. The one on the left is sharp, the right irregular. The results of processing of the raw images by edge detection operators are extremely sensitive to image noise levels; thus, images are smoothened with Gaussian and detected with Canny (Figure 8b). The boundary of the left grain is accurate, while the right is irregular. However, the edge separates the region of the right grain from the background and also sets it apart from the left grain.

Although the Canny operator has been used in numerous edge detection applications, it has two main weaknesses. First, sensitivity to image noise, which must be reduced by Gaussian smoothing. Second, false positives edges with many discontinuities, which should be removed by hysteresis thresholding. One of the first steps during edge detection with Canny is calculation of the first derivative of the image. The calculated gradient operator is sensitive to local gray level changes and can be used as an edge detector after comparison with the predefined threshold value. Besides, the non-maximum suppression algorithm is used to remove pixels that are not on the edge. Another method is calculation of the second derivative of the image by the Laplacian of a Gaussian (Figure 8c) and zero-crossing detection, which improve the detection of irregular edges. Both, first and second derivatives are sensitive to window size.

Based on the discussions regarding Figure 6, Figure 7 and Figure 8, several general problems have been identified, which have been considered in the proposed algorithm. Moreover, the authors of [48] identified three segmentation errors, especially in region growing: false object boundaries are detected by segmentation without corresponding to the real edge, real edges are not detected by the segmentation, and lack of coincidence between the segmented boundary and edge in the image. Region growing and edge detection is associated with a threshold value, which only considers the intensity of the pixel without any adjacent pixels. Thresholding typically affects the final result of segmentation by adding pixels outside or removing pixels from inside the region of interest. The final segmented region is reduced or enlarged by the extraneous pixels, which is highly inconvenient due to grain size determination. The problem of correct thresholding can be solved by using a global or local method. Global thresholding methods based on difference between classes, e.g., object and background, are significant; thus, threshold depends on the pixel’s properties (such as intensity). A commonly global thresholding method is based on the Otsu [49] algorithm. Other methods are based on local information for each pixel, such as intensity, variance, or mean in small regions such as 3 × 3 pixels. Local methods are generally sensitive to individual image characteristics. The main local techniques identified include Niblack [50], Sauvola [51], Yanowitz, and Bruckstein [52]. 

Because these methods have certain negative features, we proposed a combination of local and global information. Our main goal was accurate grain region detection; however, region growing segmentation caused limitations and excessive grain growth when attempting to identify all pixels in a grain. Therefore, we proposed a method that is based on region growing, where the process is carried to the nearest edge, followed by tracking of the edge in a closed area. As a result, all pixels between the recognized edge and the seed point from which the growth began were automatically assigned to the grain region without checking any of the thresholds.

The region-growing segmentation uses the intensity value of the pixel and a homogeneity checking procedure. While this is correct in definition, it does not work correctly in most real applications. There are four problems that should be considered: selection of the seeds (which have simply been taken from the list of pixels with the highest intensity after reducing adjacent pixels), homogeneity measure, edge detection, and size of the local neighborhood. The proposed method consists of a few main steps, as follows:

1. Input grayscale image *I*, Canny calculated binary image of edges *E*
2. Initialize threshold *T* regarding the GLCM matrix with Equation (2) and initialize list of the pixels to be searched *P* and list of the seeds *S*
3. *N* is a region number; *K* is a seed pixel number, *M* is a pixel number in a region
4. Start growing region *R_N_* from pixel *S_K_* (seed point, k = 1,..) with *T_R_* in the horizontal direction, then vertically
5. Find the edge *E_N_* based on calculations (3), (4) and (6); update *T_R_*
6. Track the edge *E_N_* with *T*
7. Assign all pixels inside the region *R_N_* between *S_K_* and the last pixel of *E_N_* to the current region; update *P* and *S*
8. If *E_N_* completed, initialize T regarding to GLCM, starting growing from *S_K_* in vertical direction
9. Repeat 5–7
10. Finish growth of region *R_N_*
11. Select next seed pixel *S*_*K*+1_ and repeat 4–10 until all the seeds from *S* are removed
12. Go to particle features’ calculation and refining process, if required

**Figure 9:**
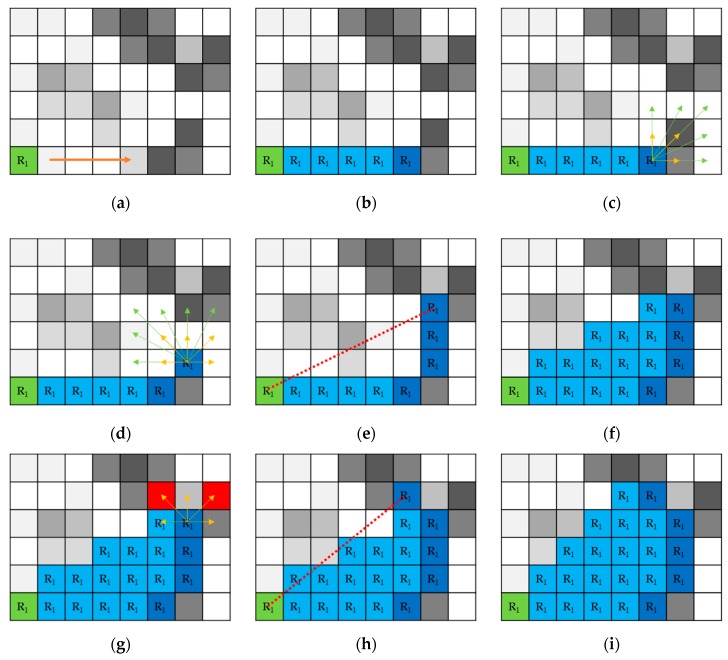
Detailed steps of the proposed algorithm. Region growing (**a**,**b**), edge tracking (**c**,**d**), region updating (**e**–**i**), selection of proper edge direction (**g**,**h**).

First, we decided to combine local and global information. The images contain a large number of grains with uniform intensity, localized in a consistent region; and small number of low-intensity pixels localized on the edges. We used Equation (3), which combines information from intensity and derivatives of the image, instead of a simple intensity value of the pixel:

(3) $$V_{N}=\sum_{1}^{q}P\left(i,j\right)-P\left(i,j\right)+f^{1}\left[P\left(i,j\right)\right]+f^{2}\left[P\left(i,j\right)\right],$$

where *q* is the number of pixels in a small neighborhood [3×3] or [5×5], *P*(*i*,*j*) is pixel intensity, *f*^1^ is a first derivative and *f*^2^ is a second derivative of image in pixel (*i*,*j*). The calculated value is then compared at each step with threshold value *T*, which is used as a homogeneity measure. At the start, the value is calculated strictly on the basis of GLCM matrix values, obtained during the quality check step. In contrast to other solutions, we updated the *T* value only when the growing region has an edge. This part of the process is presented in Figure 9a–c. 

In this case, the method skips single pixels with high intensity, but with the correct calculation of *T* can be used for edges visible in *f*^2^ detection. We decided to modify Niblack’s algorithm, which is simple and fast, but unfortunately, the value around the mean of the neighborhood of the pixel varies. We added extra information to the definition of Niblack’s threshold by consideration of Canny edge appearance in the pixel. This reduces a part of the standard deviation. Threshold is calculated as per Equation (4):

(4) $$T=\sum_{1}^{k}S\left(i,j\right)+k*\sigma \left(i,j\right),$$

where *k* is a parameter set to 0.2 for bright objects and −0.2 for dark objects in classic Niblack’s definition, *σ* is standard deviation in the neighborhood of the pixel. We used [3×3] or [5×5] neighboring regions to search for edges—the smaller one in the first round and a larger one as the second stage. We modified values for k, instead of proper edge detections:

(5) $$k=\left\{\begin{array}{l} 1,\;initialize\;stage\\ -0.2,\;if\;Canny\;edge\;detected\\ 0.2,\;if\;Canny\;edge\;not\;detected \end{array}\right\},$$

where *k* after the initialization stage depends only on the presence of edges found using an additional, corresponding Canny edge detector. Finally, the edges are detected by using the proposed consistent condition:

(6) $$V_{N}-V_{N-1}\leq T_{N-1}.$$

If Equation (6) is found to be true, then threshold *T* is updated by the value of *T_N_*. Contrary to the hysteresis method, which uses many thresholds to find the edge, we use one threshold *T* that strongly closes the area after detection of the edge. Next, this method tracks edges with defined threshold *T* in small neighboring regions of [3×3] or [5×5], when the edge is not continuous and a [3×3] edge detection fails (see Figure 9d). After each edge pixel detection, all the pixels localized in the triangle between seed point, first edge pixel, and the last one are automatically added to the region, without comparison to *T* (see Figure 9e,f). We identified diverging edges, i.e., too close grains. In Figure 9g, two pixels marked in red meet Equation (6). In this case, the Euclidean Distance *d* (Equation (7)) to seed pixel is calculated for both of them, and a pixel with the least distance is marked as an edge pixel *E_P_* (see Figure 9h):

(7) $$E_{P}=min\left\{d_{MK}\left(P_{M},S_{K}\right),d_{LK}\left(P_{L},S_{K}\right),\ldots \right\}\;where\;M\neq L,$$

This calculation ensures compactness of the grain. In Figure 9i, a sample with added edge pixel and other pixels belonging to the region of the grain is presented.

### 3.4. Particle Analysis, Refining and Classification

The proposed segmentation procedure localizes grains in the sample image. Grains, as any object, can be described by several first-, second- and third-order features. With regards to our main goal, which is determining grinding quality, two issues are of importance—the percentage distribution of fractions in the sample, and the shape quality of the grain. For this purpose, we use shape features that characterize the shape and compactness of the grain. Recent literature has found numerous shape features, a few of which are particularly useful for our method, namely, aspect ratio (*F_A_*), Heywood circularity factor (*F_H_*), and compactness factor (*Fc*). First, each detected grain is tested using *F_H_* and *Fc*, which should not be too low for correct grains; a perfect value for both is 1. A value below 0.5 suggests that the grain is probably heterogeneous; in other words, the grain consists of two or more grains that should be separated. For this reason, we used a method of refining grains, which is based on grain separation using a simple and fast distance map method. 

Finally, the grains must be classified as one of the predefined fractions. In most cases, while milling with an electromagnetic mill, we identified 4-6 fractions of different size e.g., 0.2–0.25 mm. This size is only approximate, based on grain diameter. Considering that the vision system is stationary and the distance between the camera and the sample is constant, we used a grain classification procedure that compared grain size and perimeter to the nominal values of the selected fractions. We could have used a more sophisticated classification method with weighted parameters, even without knowledge of grinded fractions; however, our system offers the possibility of comparison to nominal values—extracted using the sieves method. Finally, we are able to quantify the share of each fraction, in addition to extracting the quality of the detected grains in a statistical form.

## 4. Results and Discussion

This section presents a summary of the experiments performed above. We focus on the main steps of the algorithm, which include pre-processing, shape features’ calculation and application, grains detection core algorithm, and comparison of the proposed technique with other well-known techniques for detection of grains. We also discuss the possible use of our method to calculate the active surface of copper ore and detect the moisture levels of ground material. 

The grinding process was performed with steel rods 1–3 mm in diameter and 9–20 mm in length. With the use of laboratory sieves, copper ore grains were partitioned into five fractions: 0.045–0.071 mm, 0.071–0.1 mm, 0.1–0.2 mm, 0.2–0.5 mm, and 0.5–1 mm. Based on these sizes, the samples were tested and 500 images of different grain sizes were taken. The prepared samples were found to have uniform granularity after the screening, enabling determination of the effectiveness of the algorithm. We also performed experiments on a different set of samples, which consisted of diverse graininess, with the largest being about 0.012–0.025 mm.

**Figure 10:**
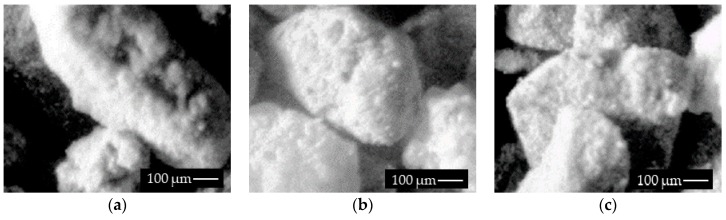
Samples of grains localization. Elongated grain (**a**), oval grain (**b**), covered grain (**c**).

In this paper, we focused on the correct detection and classification of grains to assess the grinding level of the material in each predefined granularity class. In our method, we selected dense fractions for the evaluation of separability of different granularities. In Section 3, we discussed the importance of correct illumination, because the specified technique requires a correct definition of grain boundaries and precise sample preparation. Angle illumination improves grains boundaries, increases the intensity of grains peaks, and significantly enhances the possibility of detection of correct sample surface uniformity. The 2D machine vision system used, however, has certain drawbacks, which include overlapping of grains, one over the other; and measuring of two or more inseparable grains as one individual object. These limitations persist, even on using local threshold techniques. Samples of these type of grains are presented in Figure 10; Figure 10c is a complex sample, where some grains cover parts of others.

The core of the algorithm is performed after pre-processing, which is discussed in detail in Section 3. In order to improve the quality of the grain boundaries in the sample, contrast enhancement was done, which reduced the dark areas and finally conducted the 2DFFT-GLCM cascade test. At this point, we extracted more information about grain boundary, thereby increasing the probability of a more accurate evaluation of the surface and shape features.

The identified set of shape parameters were used in two ways: refining condition and main classification. Table 1 presents a short list of the calculated parameters. While these shape features can be used to characterize the detected grains, different features have distinctive sensitivities towards shape change. The aspect ratio (*F_A_*), Heywood circularity factor (*F_H_*) and compactness factor (*Fc*) have been used as main shape features, while others have been used as additional quality measures, e.g., percentage of area/image obtain information about total segmentation quality, higher total value (means that the entire image contains a large number of grains), and smaller value (means that there is some free space between the grains). On the other hand, as presented in Table 1, the total value for large granularities is quite small, about 50%, which is a consequence of the position of grains and detected boundaries. Although the illumination was in opposite directions, we had to deal with a shadow effect in the sample. Segmentation also affects some edge points, and thus values of the shape parameters. Much smaller granularity grains are uniformly placed, with small distances between adjacent grains; thus, this parameter will be larger—about 90% for copper ore. 

**Figure 11:**
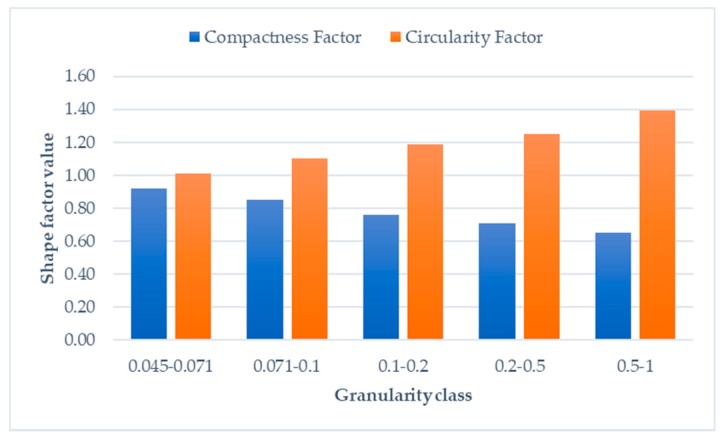
The *F_H_* and *Fc* factor for separated granularities of copper ore.

The mentioned parameters are used as detailed measures of grain shape. The values of *F_A_*, *Fc* and *F_H_* should be as close to 1 as possible. Figure 11 presents two average values of the mentioned parameters. Large granularity grains are of irregular shape, especially when their size is greater than 1 mm. During milling and grinding, the shape of the grains rapidly change, until they are more or less homogenous. They are not oval because the grains are of metal ore, as compared to sand or limestone, which is an ideal circle. The *Fc* factor is directly associated with density of the grain, whereas information about the circularity of the grain is represented by the *F_H_* factor. Both of them change during milling, starting from 1 mm and finishing at 0.045–0.071 mm.

**Figure 12:**
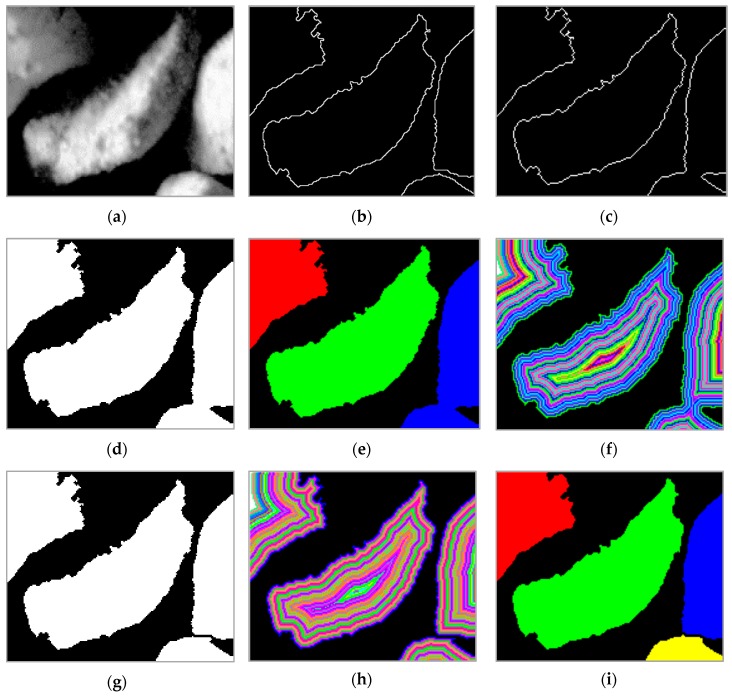
The original zoomed sample image (**a**), grains boundaries detected with the proposed algorithm (**b**), grains boundaries without the proposed modification (**c**), result of segmentation (**d**,**e**), distance map (**f**), separated grains (**g**), updated distance map (**h**), final segmentation result (**i**).

In Figure 12, we present the results of the proposed method with the modified Niblack’s threshold by including edge information. Figure 12b shows the detected grains boundaries as a result of growing and edge tracking using our proposed improvements, whereas Figure 12c presents results without improvement. 

This is visible in the sample by the lack of boundary between the two grains (visible in the right part of the image), as a result of which the segmentation could detect only three seeds (see Figure 12d,e). Particle analysis detects the suspected grain using *F_A_* and after calculating the distance map (Figure 12f), the algorithm performs separation of the grain into two individual ones. The results of this step are shown in Figure 12g,h. The updated distance map (see Figure 12i) is not automatically calculated in the algorithm; it is presented here to emphasize on the obtained result. The use of the proposed algorithm reduces the number of grains, which requires calculation of the distance map and recalculation of the shape parameters for the separated grains from 15% to 2%.

**Figure 13:**
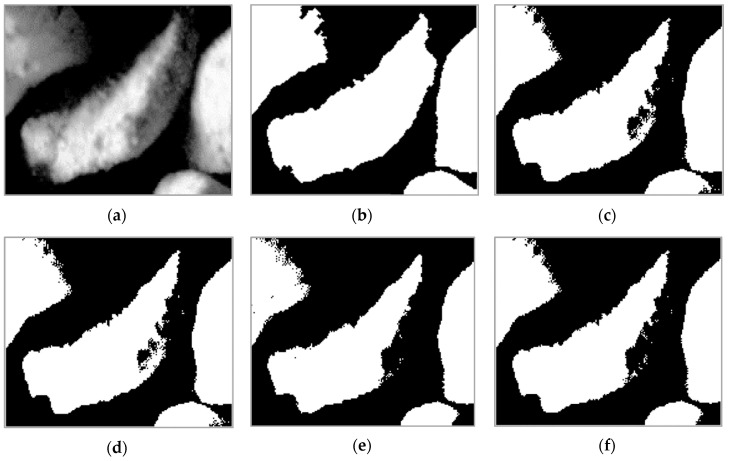
The original zoomed sample image (**a**), proposed method (**b**), global clustering (**c**), global inter-class variance (**d**), local Niblack (**e**), local Sauvola (**f**).

In this research work, we performed experiments using other methods of segmentation. The results are presented in Figure 13, where the differences between the methods are visible, especially in the area where the grains have a gradual decrease in intensity value. The shape of the grains, especially the ones in the middle, is better recognized by the proposed method (see Figure 13b) than other methods, without any additional processing (such as image morphological operations). Figure 13c,d present the results of the global thresholding methods. The clustering and inter-class variance technique that was also used produced similar results. Both of them cuts regions with low intensity, also some holes are identified inside the grain’s region. There are several numerical differences in shape features; even when only the area of the grain is taken into account, 20%–30% of the pixels are recognized as grain pixels. In contrast to the global methods, the local technique is more sensitive to rapid changes in intensity, especially in grains border regions, as presented in Figure 13e,f. The regions inside the grains are also segmented correctly as a result.

In the tested sample set, percentage of total efficiency, calculated for all the granularity classes in the sample, increased to 98.45% for the proposed method. On the other hand, manual selection obtained about 5000 pixels more. In Table 2, a comparison of the selected parameters for a reference, Otsu segmentation, and proposed method is presented; in this case, for fraction 0.1–0.2 mm, where manual selection was possible. Generally, it is the number of grains that are taken into account; however, quality assessment is based on more adequate measures, as presented in Table 2.

**Figure 14:**
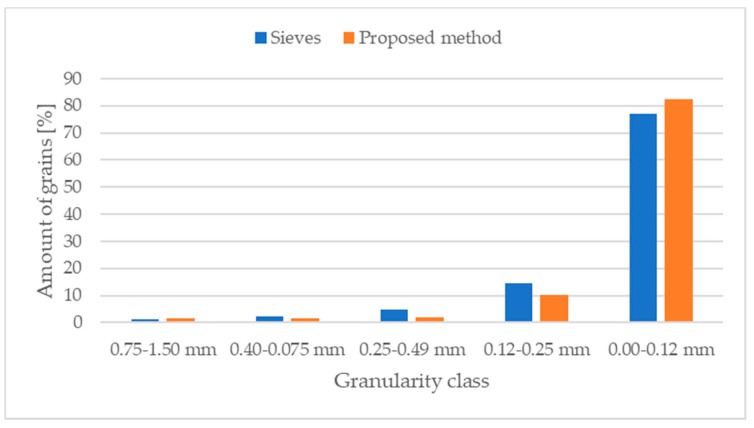
The comparison of total amount of grains [%] in the proposed and reference methods.

**Figure 15:**
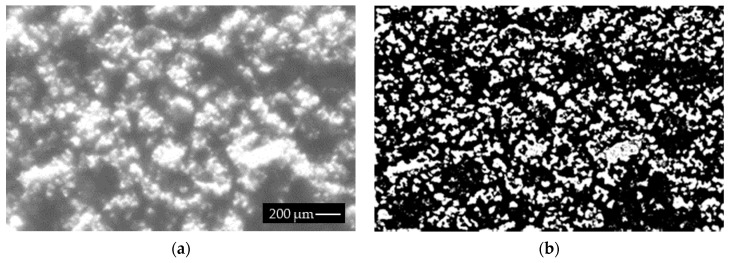
The acquired images of mixed granularities (**a**), results of the proposed method (**b**).

We also carried out a set of experiments for samples with a variable composition of fractions, although with a dominant fraction. Each sample had five fractions, with the 0.00–0.12 mm fraction taking the highest share of almost 80%, followed by the 0.12–0.25 fraction with 15%, and the remaining three grain fractions accounting for 5%. The experiments focused on the sample with a large share of small granularity classes, in order to check the efficiency of the method to detect and separate grains in small, closed classes. As the reference method used laboratory sieves, which can also be used by other methods to evaluate granularity, this comparison dealt with some inaccuracies caused by the square shape of the aperture in contrast to the irregular shape of the real grains. A comparison between the sieves and proposed methods is presented in Figure 14, where the latter achieved correct results. For the granularity classes, this method achieved the following errors: −0.24, 0.71, 2.9, 4.32, −5.42, which agree with our machine vision system parameters (we selected granularity of about 80-100 µm for correct detection). Figure 15a presents the image of the tested sample, while Figure 15b shows the segmentation results. Although the results are accurate, the sample preparation method has one drawback: conglomerates of small grains are formed for small granularities of copper ore and materials such as gravel, limestone and other metal ores.

Despite the fact the algorithm produces good results and takes about only 350 ms (20% for pre-processing and 80% for main algorithm) on an INTEL i5 2.60 GHz PC computer, it has certain drawbacks, as does the machine vision/image processing. First, the sample preparation process offers weak results, even with manual segmentation, mainly by creating dark areas, which then result in loss of pixel information. Second, illumination causes incorrect lighting angle and positioning, which affects images in bright areas and shows unrecognized grains. This is also related to boundary detection, where multiple grains might appear as one. Next, grains are randomly positioned in the sample and the size of measured grains might not be a correct representation of their three-dimensional shape. This problem also occurs when the sieve technique is used, especially for elongated grains. 3D image processing can generally handle this problem. Finally, in comparison to a light microscopy system especially for very small granularities, determination of the shape of the grains is not as accurate as the microscopy method regarding the air fluctuation in spacer rings tube, camera resolution and lenses limitations. The angle illumination will not extract boundaries for microscopic granularities. Thus, in most cases, we have hardware limitations, instead of a software algorithm, which can be adapted for microscopic images analysis.

## 5. Conclusions

This paper presents a method of copper ore grain detection and classification. The authors have developed a new method by combining local and global information of grains edges. In the proposed calculation of parameter *V_N_*, the authors combined information from the intensity, and first and second derivatives of the image, instead of a simple intensity value of the pixel. Next, the modified Niblack’s threshold and procedure to update the current threshold is described in detail. The authors discussed the problems related to detection and classification of copper ore and a detailed analysis of shape features of detected grains was performed, based on a few well-defined parameters, which include homogeneity, circularity, aspect ratio, and area factors. 

This research focused on different granularity mixture materials with predefined granularity classes. The obtained results show the relationship between measured shape features and overall quality of the milling process. A comparison of results from the image analysis and those from screening technology yields good results. One of the main advantages of our method is a wide range of applications, such as detection and tracking of a selected fraction with large grains, and detection and tracking of a few fractions with different grains sizes. The proposed method is a rapid, reliable and robust technique for grain-size analysis and an inexpensive alternative to screening technology and vibration measurements. This method has the advantage of being automated and objective in electromagnetic mill installation.

In our next research study, we aim to focus on the detection of the active surface of copper ore, and determination of the relationship between material moisture and granularity. In our experiments, we investigated the active surface of copper ore, which is the most important parameter of a flotation process. This surface can be detected by correct adjustment of illumination and aperture of the lens. It is possible to acquire an image that clearly shows the active surface using a color camera for copper-colored areas. Detection of these areas can be carried out by using the developed algorithm, with seeds selected from pixels in the copper-colored areas. The relationship between material moisture and granularity class is based on study of the wet grinding process, where moisture causes conglomeration of smaller grains, thus affecting the final results of the detection process. The overall granularity decreases during grinding; thus, the detection of large grains, instead of only small ones, can be used in a moisture control system.

## Figures and Tables

**Table 1:** Selected shape features for Figure 7d.

Shape Feature/Object No.	1	2	3	4
Perimeter [Pixels]	253	444	267	127
Elongation Factor	2.84	3.24	4.64	3.79
Compactness Factor (*Fc*)	0.57	0.39	0.79	0.69
Circularity Factor (*F_H_*)	1.39	1.55	1.39	1.31
Aspect Ratio (*F_A_*)	0.86	0.59	0.95	0.94
Convex Hull Perimeter [pixels]	233.95	396.24	265.39	124.72
Max Feret Diameter [pixels]	92.03	166.90	118.23	54.23
Area [Sq. Pixels]	2657	6543	2943	748
Orientation [°]	82.89	39.06	87.60	3.07
Area/Image Area [%]	9.72	23.94	10.77	2.74

**Table 2:** Selected shape features for Figure 7 with fraction 0.1–0.2 mm.

	Manual Selection	Adaptive Segmentation	Proposed Method
Number of grains	76	69	77
Sum of grains area [pixels]	402,639	358,325	397,186
Average compactness factor (*F_C_*)	0.70	0.65	0.68
Average circularity factor (*F_H_*)	1.14	1.21	1.15
Total efficiency [%]	-	91.39	98.45
